# FG020326 Sensitized Multidrug Resistant Cancer Cells to Docetaxel-Mediated Apoptosis via Enhancement of Caspases Activation

**DOI:** 10.3390/molecules17055442

**Published:** 2012-05-09

**Authors:** Xiu-Wen Wang, Xiao-Kun Wang, Xu Zhang, Yong-Ju Liang, Zhi Shi, Li-Ming Chen, Li-Wu Fu

**Affiliations:** State Key Laboratory of Oncology in Southern China, Cancer Center, Sun Yat-Sen University of Medical Sciences, Guangzhou 510060, China; Email: wnwnwang@163.com (X.-W.W.); wxk198708@163.com (X.-K.W.); zsdxzhangxu@yahoo.com.cn (X.Z.); liangyju@mail.sysu.edu.cn (Y.-J.L.); shizhi81@yahoo.com.cn (Z.S.); chlm78@yahoo.com.cn (L.-M.C.)

**Keywords:** cancer, multiple drug resistance, FG020326, P-glycoprotein, apoptosis, caspase

## Abstract

Apoptotic resistance is the main obstacle for treating cancer patients with chemotherapeutic drugs. Multidrug resistance (MDR) is often characterized by the expression of P-glycoprotein (P-gp), a 170-KD ATP-dependent drug efflux protein. Functional P-gp can confer resistance to activate caspase-8 and -3 dependent apoptosis induced by a range of different stimuli, including tumor necrosis and chemotherapeutic drugs such as docetaxel and vincristine. We demonstrated here that comparison of sensitive KB cells, P-gp positive (P-gp^+ve^) KBv200 cells were extremely resistant to apoptosis induced by docetaxel. FG020326, a pharmacological inhibitor of P-gp function, could enhance concentration-dependently the effect of docetaxel on cell apoptosis and sensitize caspase-8, -9 and -3 activation in P-gp overexpressing KBv200 cells, but not in KB cells. Therefore, the enhancement of caspase-8, -9 and -3 activation induced by docetaxel may be one of the key mechanisms of the reversal of P-gp mediated docetaxel resistance by FG020326.

## Abbreviations

MDR: multidrug resistance
P-gp: P-glycoprotein
P-gp^−ve^: P-gp negative
P-gp^+ve^: P-gp positive
PARP: poly-(ADP-ribose) polymerase
PBS: phosphate-buffered saline
fmk: fluoromethylketone
GB: granzyme B
pfp: perforin

## 1. Introduction

Docetaxel (Taxotere, TXT), a semisynthetic antineoplastic agent that is very similar to paclitaxel in structure, mechanism of action, and spectrum of antitumor activity, differing structurally from paclitaxel at the C-10 position where docetaxel has a hydroxy group instead of an acetyl group and contains an OC(CH_3_)_3_ moiety on the C-13 side chain as opposed to a benzamide phenyl group as in paclitaxel, has significant antitumor activity in a number of human cancers, including advanced ovarian, breast and non-small cell lung carcinomas. Docetaxel enhances the assembly of stable microtubules from tubulin dimers and inhibits their depolymerization. Incubation of cells with docetaxel leads to the formation of abnormal bundles of microtubules and results in the arrest of cells in the G2/M phase of the cell cycle [1]. However, biochemical events downstream of docetaxel binding to microtubules, which lead to apoptosis, are not well understood. Recent discoveries of activation of signaling molecules by paclitaxel and paclitaxel-initiated transcriptional activation of various genes indicate that paclitaxel initiates apoptosis through multiple mechanisms. It is involved in p53 status, bcl-2 phosphorylation and the activation of JNK/SAPK and p34^cdc246^ and caspase-3 [2,3,4,5,6,7]. Docetaxel, a taxane derivate, promoted the formation of reactive oxygen species (ROS) in mitochondria and elicited reduction of mitochondrial membrane potential, and release of cytochrome c to cytosol, and activated caspase-9 and -3 [8].

Multidrug resistance mediated by the drug efflux protein, P-glycoprotein (P-gp), is one of the mechanisms that tumor cells use to escape death induced by chemotherapeutic agents such as paclitaxel and docetaxel. As a member of the adenosine triphosphate-binding cassette transporter family, P-gp has traditionally been thought to confer resistance to chemotoxins by actively effluxing them from the cells [9]. Several noncytotoxic drugs could bind with P-gp and inhibit its function. This inhibition can sensitize MDR cells to chemotherapeutic drug *in vitro* and *in vivo* [10,11,12,13]. They included calcium channel blockers (e.g., verapamil, nifedipine), calmodulin antagonists (e.g., trifluoperazine, chlorpromazine), various steroids (e.g., progesterone, tamoxifen), quinolines (e.g., chloroquine, quinidine), immunosuppressive drugs (e.g., cyclosporine, PSC-833, rapamycin), antibiotics (e.g., rifapicin, tetracyclines), surfactants (e.g., tween 80, cremophor EL), and yohimbine alkoids (e.g., reserpine, yohimbine), all of which have been shown to reverse MDR *in vitro* and/or *in vivo*. The mechanism of action of P-gp has been investigated extensively from a biochemical perspective in the past few years. The use of purified P-gp and functionally reconstituting it into liposomes to investigate its properties show that P-gp alone is sufficient to transport different drugs [14]. The decrease of cellular anticancer drug accumulation was due to the increase of extrusion of drug mediated by P-gp.

Importantly, the reports by Johnstone *et al*. [15,16] and Robinson *et al*. [17] have indicated that, in addition to its role as an efflux pump, P-gp regulates programmed cell death induced by antineoplastic agents, serum starvation, UV irradiation, and ligation of the cell surface death receptors Fas and tumor necrosis factor (TNF) receptor. Generally, these diverse apoptotic stimuli are dependent on the activation of caspases to initiate cell death. Johnstone *et al.* [16] demonstrated that functional P-gp inhibited activation of caspase -8 and -3 following Fas ligation and this inhibitory effect could be reversed by inhibiting P-gp, such as using specific anti-P-gp monoclonal antibodies (mAbs). Many chemotherapeutic drugs, such as doxorubicin and vincristine, induced cell apoptosis in a receptor-dependent manner [15,16,18,19]. Therefore, P-gp may play a dual role in regulating cell death induced by these stimuli by (i) removing the toxins from the cell and (ii) by inhibiting the activation of caspases.

Our previous experiments showed that FG020326, 2-[(4-Methyl-*trans*-acrylate)phenyl]-4,5-bis-(4-*N*,*N*-methylisopropylaminophenyl)-1(*H*)-imidazole, produced a potent MDR reversal *in vitro* and *in vivo* and increased the accumulation of rhodamine and doxorubicin (Dox) in MDR cells [10]. To investigate whether FG020326 could reverse the apoptotic resistance to docetaxel and whether it is involved in apoptotic mechanisms, DNA fragmentation and the pathway of apoptosis induced by docetaxel were studied in the presence or absence of FG020326 in P-gp^+ve^ KBv200 cells and their parental P-gp^−ve^ sensitive KB cells.

## 2. Results and Discussion

### 2.1. KBv200 Cells with Overexpression of P-gp Are Resistant to Docetaxel-Mediated Death

**Figure 1:**
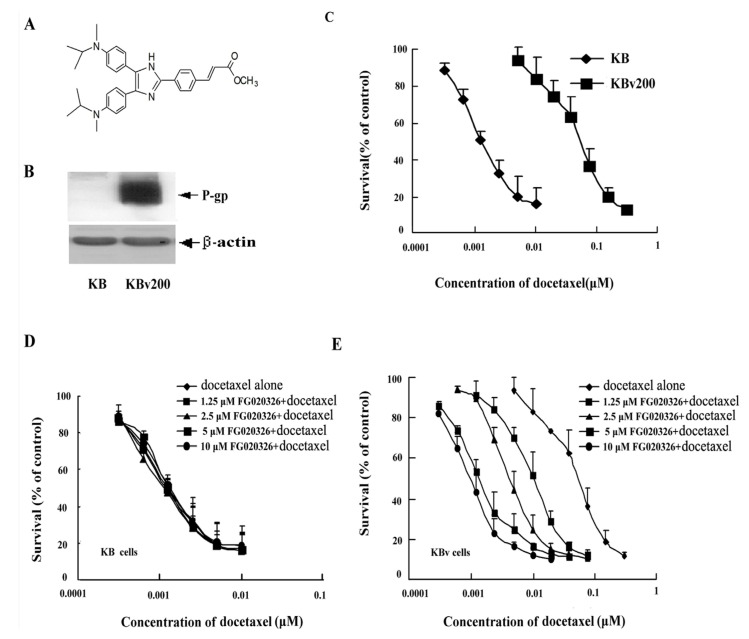
(**A**)The structure of FG020326; (**B**) The overexpression of P-gp detected by western bolt in KB cells and KBv200 cells. The P-gp was overexpressed in KBv200 cells; (**C**) The cytotoxicity of docetaxel in KBv200 and KB cells. The cells were cultured with a full range of concentrations of docetaxel for 72 h. Data represent means and standard errors of at least a triplicate determination; (**D** and **E**)Effect of FG020326 on enhancing the sensitivity of KB cells and KBv200 cells to the chemotherapeutic agent *in vitro*. Cytotoxicity was measured using a MTT assay. The cells were cultured with a full range of concentrations of docetaxelin the presence or absence of FG020326 for 72 h. Data represent means and standard errors of at least triplicate determinations.

KBv200 cells, a classical multidrug resistant human epidermoid carcinoma cell line that expressing high levels of P-gp, were cloned from parental drug-sensitive KB cells by stepwise exposure to increasing doses of vincristine and ethylmethane sulfonate (EMS) mutagenesis (Figure 1B). KB cells or KBv200 cells were cultured in the presence of various concentration of docetaxel for 72 h, and cell death was determined by MTT assay. As shown in Figure 1C, P-gp^−ve^ KB cells were effectively killed by the chemotherapeutic agent, docetaxel (IC_50_: 1.3 ± 0.2 nmol/L), whereas P-gp^+ve^ KBv200 cells were resistant to docetaxel-induced death (IC_50_: 69.8 ± 7.3 nmol/L). KBv200 cells were approximately 54-fold resistant to docetaxel compared to the parental sensitive KB cells in our experimental system.

### 2.2. Effect of FG020326 on the Reversal of MDR

The cells were incubated with various concentrations of FG020326 and a full range of concentrations of the chemotherapeutic agent docetaxel. The aim of the experiments was to see if FG020326 changed the sensitivity of the cells to docetaxel. FG020326 at concentrations of 10.0, 5.0, 2.5 and 1.25 μmol/L, which are barely cytotoxic (more than 90% cell survival) to the KB and KBv200 cells, lowered the IC_50_ of docetaxel to 1.5, 2.4, 6.5 and 17.3 nmol/L in the KBv200 cells. This gave a 46.5, 29.1, 10.7 and 4.0-fold reversal of MDR, respectively. These results suggested FG020326 was very effective at reversing MDR *in vitro*. In the parental sensitive KB cells, the IC_50_ of docetaxel was 1.4, 1.3, 1.3 and 1.4 nmol/L in the presence of FG020326 at concentrations of 10.0, 5.0, 2.5 and 1.25 μmol/L, respectively. These results suggested that FG020326 had no effect on KB cell sensitivity to docetaxel (Figure 1D,E, Table 1).

### 2.3. P-gp^+ve^ KBv200 Cells Are Resistant to Docetaxel-Mediated Apoptosis

**Figure 2:**
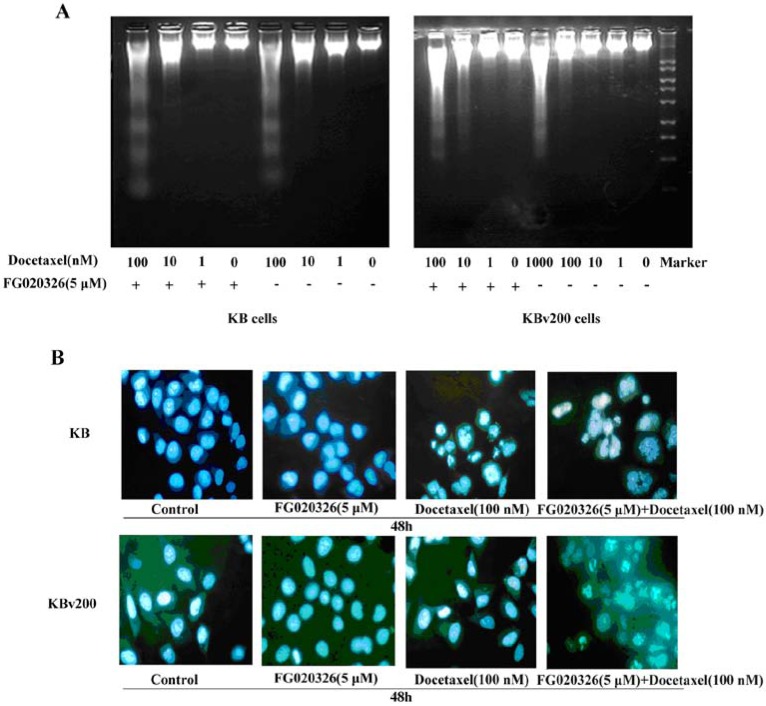
(**A**) DNA fragmentation in KB and KBv200 cells exposed to docetaxel in the presence or absence of FG020326. DNA obtained from KB and KBv200 cells exposed to various concentration docetaxel for 48 h in the presence or absence of 5 μM docetaxel, respectively; (**B**) Apoptotic morphology of KB and KBv200 cells (×400) by Hoechst 33258. Cells were treated with docetaxel in the presence or absence of FG020326. The cells were stained by Hoechst 33258, apoptotic cells were identified by their typical appearance of condensed and fragmented nuclei.

Previously, it has been reported that docetaxel induced apoptosis in several cancer cell lines [2,3,4,5]. To characterize the biochemical and morphological changes in cells undergoing docetaxel-induced cell death, KB and KBv200 cells were cultured with docetaxel in the presence or absence of 5 μmol/L FG020326 for 48 h and subsequently assayed for classical hallmarks of apoptosis, such as chromatic condensation, DNA laddering. Treatment of P-gp^−ve^ KB cells with 100 nmol/L of docetaxel resulted in DNA fragmentation as evidenced by the formation of DNA ladders (Figure 2A). In addition, KB cells treated with docetaxel and subsequently stained with Hoechst 33258 displayed chromatin condensation and fragmentation indicative of apoptotic nuclei (Figure 2B).

**Table 1:** Enhancement of sensitivity to docetaxel (TXT) induced by FG020326 in KB and KBv200 cells. The IC_50_ of docetaxel was determined in the presence of various concentrations of FG020326. Each value represents the mean±standard deviation (S.D.) of at least three independent experiments. The fold-reversal of MDR is defined as the ratio of the IC_50_ for docetaxel alone versus the IC_50_ for docetaxel in the presence of the modulating agent.

Concertration of FG020326 (μM)	IC_50_ of docetaxel (μM)) ± SD		Fold-reversal of MDR	
	KB	KB_V_200	KB	KB_V_200
0	1.3 ± 0.2	69.8 ± 7.3	-	-
10	1.4 ± 0.5	1.5 ± 0.4 **	0.87	46.53
5	1.3 ± 0.4	2.4 ± 1.8 **	1.00	29.07
2.5	1.3 ± 0.2	6.5 ± 5.6 **	0.99	10.70
1.25	1.4 ± 0.3	17.3 ± 11.4 **	0.87	4.03

** represent *p* < 0.01 for values *versus* that obtained in the absence of FG020326.

Interestingly, P-gp^+ve^ KBv200 cells typically displayed much less nuclear fragmentation and chromatin condensation (Figure 2B) than P-gp^−ve^ KB cells following the same concentration of docetaxel. Moreover, FG020326 significantly increased cell nuclear fragmentation and chromatin condensation induced by docetaxel in KBv200 cells, but not in KB cells (Figure 2). These results exhibited that FG020326 could enhance docetaxel-mediated cell apoptosis in KBv200 cells, which appeared DNA fragmentation in 100 nmol/L docetaxel in the presence of 5 μmol/L FG020326; but not in KB cells (Figure 2A). These suggested FG020326 reversed KBv200 cell resistance to docetaxel-mediated cell apoptosis.

To further confirm the reversal effect of FG00326 on docetaxel-mediated apoptosis in MDR cells, KB and KBv200 cells were treated with docetaxel for various time periods (12, 24 and 48 h) in the presence or absence of FG020326 of 5 μmol/L, and the cell apoptosis was analyzed by flow cytometry. When treatment with docetaxel (10 nmol/L), KB cells significantly increased time-dependently in the sub-G1 phase, indicative of apoptotic peaks.

**Figure 3:**
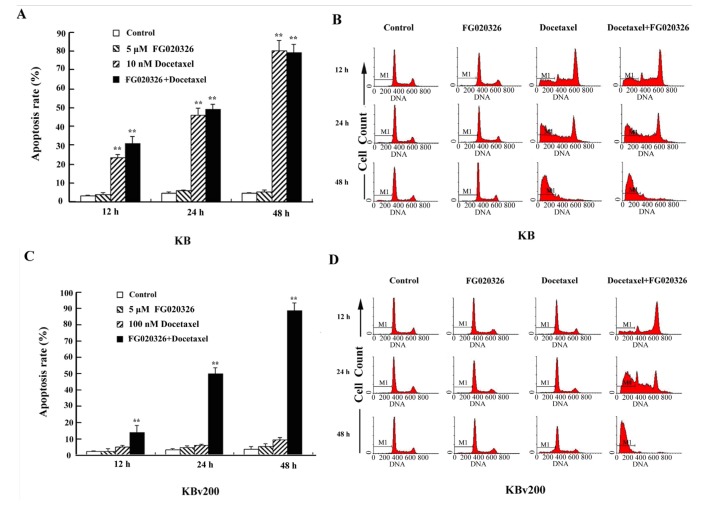
Flow cytometry analyzed the apoptosis-induced by docetaxel in the time-dependent manner in the presence or absence of FG020326.The cells were treated with 5 μmol/L FG020326 or (**A**,**B**: 10 nmol/L docetaxel for KB cells) or (**C**,**D**: 100 nmol/L docetaxel for KBv200 cells) or the combination of docetaxel and FG020326 for 48 h, the sub-G1 phase cells were identified as apoptosis cells showed as M1.

The treatment of combination with docetaxel and FG020326 did not significantly increase apoptotic cells in same time point compared with treatment of docetaxel alone in KB cells (Figure 3A,B). On the other hand, KBv200 cells remarkably increase in the sub-G1 phase in time-dependent manner after treatment of 100 nmol/L docetaxel in the presence of FG020326 compared with the absence of FG020326 (Figure 3C,D).

### 2.4. The Activation of Caspase-8, -9 and -3 Are Involved in Cell Apoptosis Induced by Docetaxel

**Figure 4:**
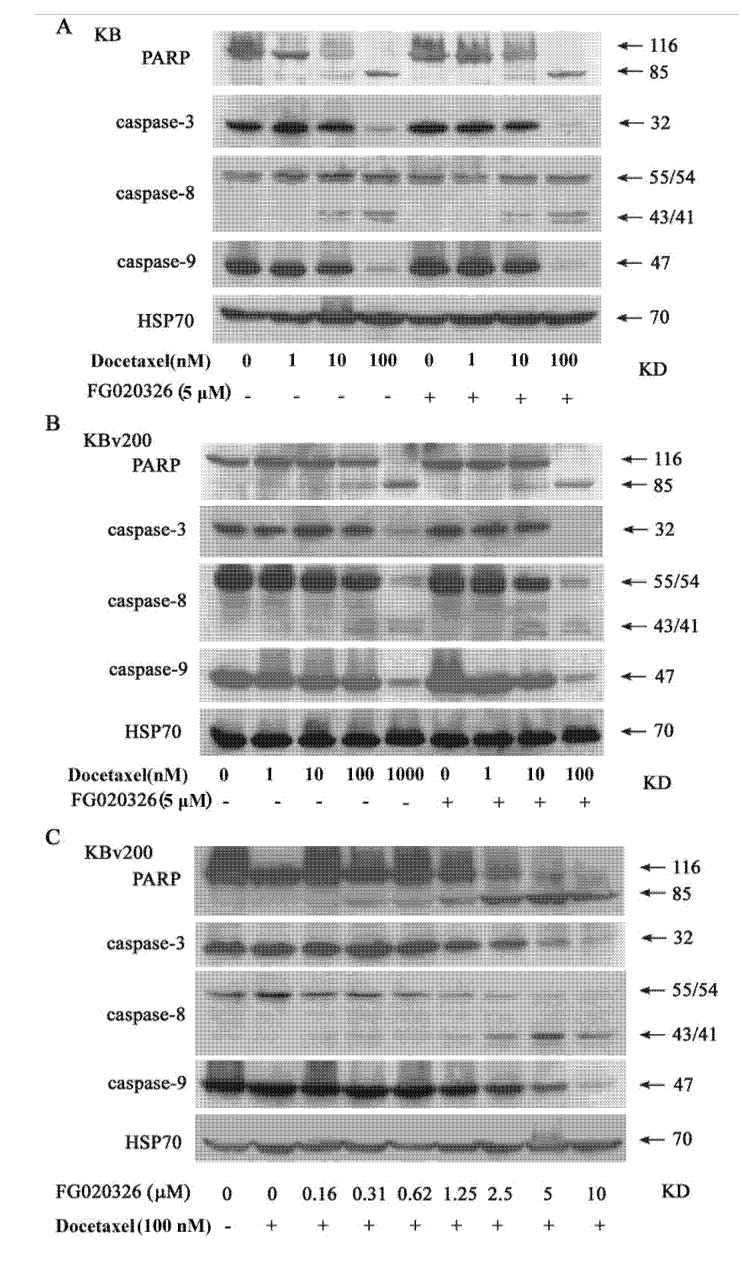
Cleavage of caspase-8, -9, -3 and PARP were showed in KB cells (**A**) and KBv200 cells (**B**) treated by docetaxel in the presence or absence of FG020326. KB and KBv200 cells were treated with various concentration docetaxel in the presence or absence of5 μmol/L FG020326 for 48 h. Cleavage of caspase-8, -9, -3 and PARP was detected by western blot. FG020326 enhancedthe docetaxel-induced caspases and PARP cleavage in concentration-dependent manner; (**C**)KBv200 cell were treated with 100 nmol/L docetaxel in various concentration FG020326 for 48 h. And then proteins were drawn and western blot was performed as described in “materials and methods”.

To examine the signal transduction pathway of apoptosis leading to DNA fragmentation, caspase-8, -9 and caspase-3 were determined by Western blot after the cells were treated with different concentrations of docetaxel for 24 h. These results showed that caspase-8 was cleaved and caspase-9, -3 degraded in docetaxel-treated KB and KBv cells (Figure 4A,B). Interestingly, Caspase-8 was cleaved and activated in the treatment with 100 nmol/L docetaxel for 24 h in KB cells, but the change in P-gp^+ve^ KBv200 cells was not observed until treated with 1 μmol/L of docetaxel. And the degradation of caspase -9, -3 revealed the similar trend (Figure 4A,B). These results suggested that caspase-8, -9 and -3 may play a key role in docetaxel-mediated apoptosis. Also, P-gp may protect cell from apoptosis induced by the activation of caspase-8, -9 and caspase-3 originated from docetaxel.

### 2.5. FG020326 Could Overcome Resistance to Docetaxel-Mediated Apoptosis via the Pathway of Sensitizing Activation Of Caspase-8, -9 and -3 in P-gp^+ve^ KBv200 Cells

Multidrug-resistant KBv200 cells and parental sensitive KB cells were used to investigate that FG020326 sensitized activation of caspase-8, -9 and -3 induced by docetaxel. KBv200 cells, with an overexpression of P-gp (Figure 1B), are resistant to docetaxel-induced DNA fragmentation in comparison with P-gp^−ve^ KB cells (Figure 2A). FG020326, a pharmacological inhibitor of P-gp, did not activate caspase-8 and -3 and induce cell apoptosis by itself in KB and KBv200 cells, but sensitized activation of caspase-8, -9 and -3 mediated by docetaxel in KBv200 cells (Figure 4A,B). These results exhibited that caspase-8, -9 and -3 did not activate until treated with 1,000 nmol/L docetaxel in KBv200 cells, but 5 μmol/L of FG020326 could sensitize caspase-8, -9 and -3 activation induced by docetaxel (100 nmol/L) in KBv200 cells, while showed no effect in KB cells (Figure 4A,B).

**Figure 5:**
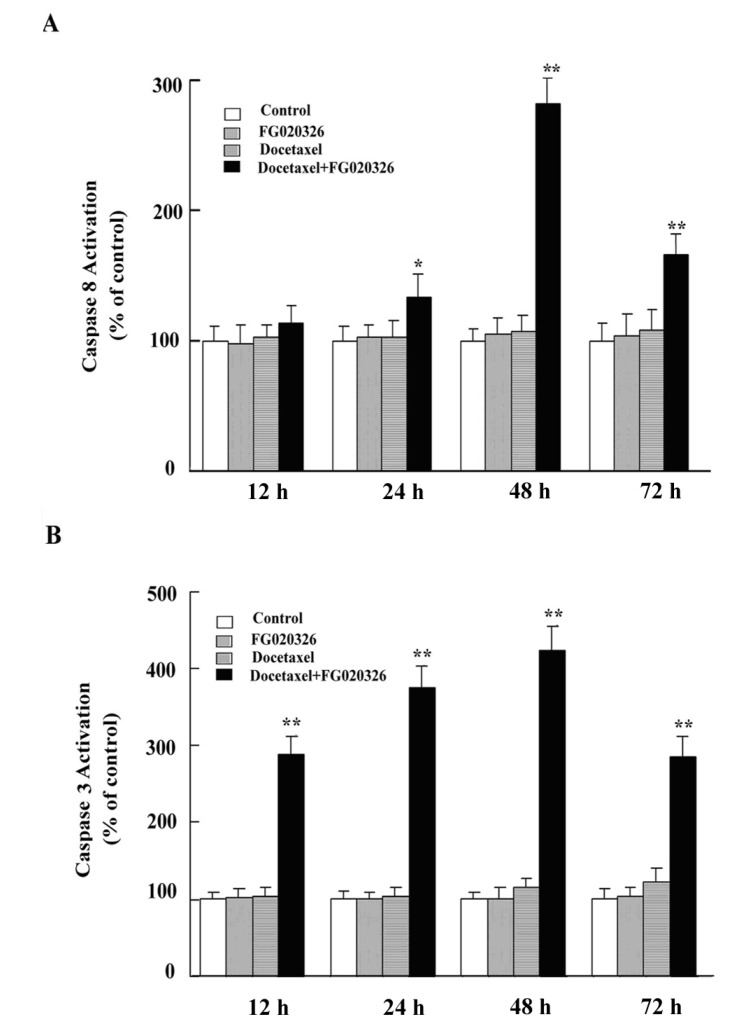
FG020326 sensitized caspase-8 (**A**) and -3 (**B**) activation induced by docetaxel.

In addition, caspase-8 and -3 activity assay showed similar results (Figure 5). The data implied that FG020326 might overcome P-gp-mediated apoptotic resistance via enhancing the sensitivity of caspase-8, -9 and -3 activated by docetaxel.

To further confirm whether the reversal of docetaxel-mediated apoptotic cell death by FG020326 was involved with caspase-8, -9 and -3, the experiments of concentration-dependent FG020326 increasing the sensitivity of caspase-8, -9 and -3 activation and apoptosis induced by docetaxel were performed. The results showed that docetaxel alone could not induce the cleavage of PARP and activation of caspase-8, -9 and -3 in KBv200 cells at the concentration of 100 nmol/L, but the combination of 100 nmol/L docetaxel with FG020326 concentration-dependently activated caspase-8, -9 and -3 and induced cell apoptosis in KBv200 cells (Figure 4C).

### 2.6. Discussion

Apoptosis is a major form of cell death characterized by a series of stereotypic morphological features. It occurs in two phases, an initial commitment phase followed by an execution phase involving the condensation and fragmentation of nuclear chromatin, dilation of the endoplasmic reticulum, and alterations to the cell membrane resulting in recognition and subsequent phagocytosis of the cells [20,21]. Apoptotic cell death is triggered by intracellular cues such as DNA damage and osmotic stress, and extracellular cues including growth factor withdrawal, matrix detachment, and direct cytokine-mediated killing. Two central pathways are involved in the process of apoptotic cell death, one involving the activation of the caspase proteases and a second, mitochondrial pathway [22].

Mitochondrial pathway involves the disruption of the mitochondrial transmembrane potential and the release of mitochondrial proteins such as cytochrome c [23,24,25] and apoptosis-inducing factor [26,27]. Cytochrome c cooperates with dATP and Apaf-1 to induce the activation of caspase-9 that can cleave and activate caspase-3 [28,29]. Importantly, release of mitochondrial cytochrome c is inhibited by anti-apoptotic members of the Bcl-2 family, such as Bcl-2 and Bcl-XL, and promoted by pro-apoptotic members such as Bid, Bax, and Bak [30,31]. No evidence ascertains P-gp is involved in mitochondrial dependent apoptotic pathway.

Caspases’ sequential activation is one of the major pathways of apoptosis induced by chemotherapeutic drugs such as paclitaxel, vincrinstine and doxorubicin. Intracellularly, caspases, a family of cysteine proteases, exist as inactive zymogens (procaspases) that have NH_2_-terminal prodomains plus large and small catalytically active subunits. Caspases play a critical role in the execution phase of apoptosis and are responsible for many of the biochemical and morphological change associated with apoptosis. It has been proposed that “initiator” caspases with long prodomains, such as caspase-8, -10, and -2, either directly or indirectly activate “effector” caspases, such as caspase-3, -6, and -7. These effective caspases then cleave intracellular substrates, such as poly (ADP-ribose) polymerase (PARP) and lamins, during the execution phase of apoptosis. As caspase-8 can activate many known caspases such as caspase-3 *in vitro*, it is a prime candidate for an initiator caspases in many forms of apoptosis [21,32,33,34,35,36]. P-gp can protect cell from apoptosis induced by the change of caspase-8 and -3 activated by chemotherapeutic drugs.

Caspase-3 cleaving PARP is the core of the apoptotic pathway induced by either anticancer drugs or receptors. But the caspase-3 activated by the first apical caspase, either caspase-9 or caspase-8, depends on various stimuli. Caspase-8 is very important in apoptosis induced by chemotherapeutic drugs. Apoptotic signals induced via membrane of the TNF receptor family, such as the CD95/Fas receptors, recruit CD95 receptor-associated adaptor proteins with death domains of caspase-8, leading to the activation of caspase-8. The active caspase-8 directly or indirectly activates “effector” caspase, such as caspase-3, -6, and -7 [32,33].

Docetaxel elicited ROS production from NADPH oxidase, which in turn triggered activation of caspase-3, leading to apoptosis in HL-60 cells [37]. Docetaxel promoted the formation of reactive oxygen species (ROS) in mitochondria, and preincubation of cells with anti-oxidants such as *N*-acetyl cysteine and pyrrolidine dithiocarbamate, protected against apoptosis mediated by docetaxel. Furthermore, treatment with docetaxel elicited reduction of mitochondrial membrane potential, and release of cytochrome c to cytosol, after 48 h of treatment. Taniguchi *et al. *[8] observed binding activity to NF-kappaB consensus site and interference with the mitochondrial function via NF-kappaB after treatment with docetaxel. Preventing pro-apoptotic property of NF-kappaB inhibited docetaxel-induced apoptosis. Thus, these results suggest that, following the activation of NF-kappaB by docetaxel, apoptosis is elicited through a mitochondria-dependent pathway. FG020326 could increase the intracellular accumulation of doxorubicin in KBv200 cells [10]. Therefore, FG020326 could also increase the intracellular accumulation of docetaxel in KBv200 cells because docetaxel is a substrate of P-gp like doxorubicin.

Caspase-8 is the most apical caspase in Fas-induced apoptosis. The cells of mice lacking caspase-8 are resistant to apoptosis induced by the TNF receptors CD95 and DR3 [38,39]. The exposure of KB cells to docetaxel increased the activity of apical caspase-8 and of effector’s caspase-3. These results suggested that caspase-8 and -3 activation was an important pathway of apoptosis induced by docetaxel in KB cells. P-gp, which is responsible for MDR, has been demonstrated to protect human tumor cells from death induced by multiple chemotherapeutic drugs. Recently, Smyth *et al.* [15] have shown P-gp^+ve^ cells could escape from the apoptosis induced by Fas. This suggested P-gp could protect from apoptosis induced by caspase-8 as the most apical caspase. Robinson *et al. *[17] have shown that P-gp-expressing cells produced by MDR1 gene transfer are resistant to apoptosis induced by serum deprivation and others of cell death stimuli. P-gp antibody such as MRK-16 or UIC2, a P-gp functional inhibitor, can reverse the resistance to apoptosis induced by Fas or chemotherapeutic drug such as vincristine [15,16]. However, Johnstone *et al.* have shown that cell apoptosis mediated by cell membrane lysis induced by perforin (pfp)/granzyme B (GzB) is via the pathway which is not involved with caspase-8 as the most apical caspase. Therefore, P-gp did not protect from cell death induced by pfp/GzB [22]. This implied that P-gp could not protect from cell death induced by the pathway of independent caspase-8 as the initiator.

KBv200 cells displayed a high-level expression of P-gp and were less sensitive to a spectrum of chemotherapeutic drugs than their parental sensitive KB cells [40]. Our experiments showed that KBv200 cells were resistant to docetaxel that mediates DNA fragmentation via the pathway of a caspase-8 as the initiator. FG020326 could not activate caspase-8 and caspase-3 and induce cell apoptosis by itself. FG020326 also did not decrease the concentration of docetaxel for fragmenting DNA and activating caspase-8 and -3 in sensitive KB cells. These suggested FG020326 did not affect apoptosis induced by docetaxel in drug sensitive KB cells. Importantly, the DNA of drug-resistant KBv200 cells could be fragmented by 100 nmol/L docetaxel in the presence of FG020326, P-gp functional inhibitor, which did not result in apoptosis alone. Inhibition of P-gp function, with FG020326, resulted in cleavage and activation of pro-caspase-8, -9 and -3 in lower concentrations of docetaxel (100 nmol/L) in drug-resistant KBv200 cells. These suggested that the circumvention of apoptotic resistance to docetaxel by FG020326 was involved in caspase-8, -9 and -3 activation in KBv200 cells.

**Figure 6:**
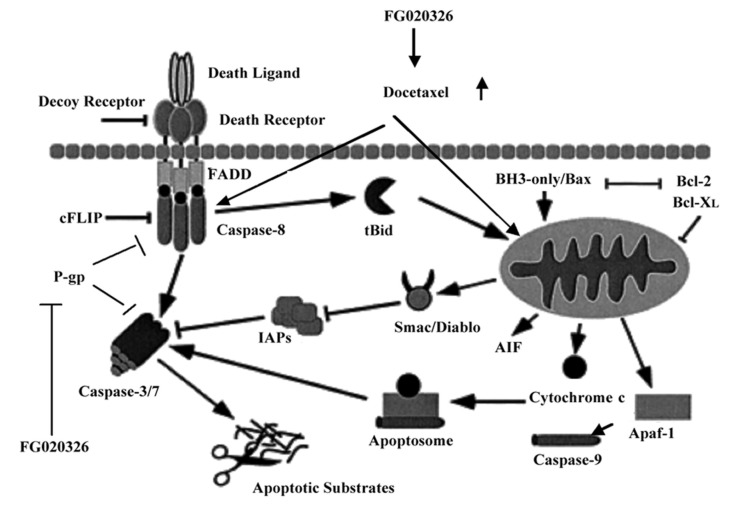
The mechanisms of FG020326 on the reversal of apoptotic resistance. (1) FG020326 could reverse MDR by inhibiting P-gp transporter function and increasing intracellular accumulation of docetaxel; (2) FG020326 might also regulate apoptosis induced by docetaxel by sensitizing the activation of caspase-8, -9 and caspase-3.

Although no evidence showed P-gp was involved in caspase-9 activation, caspase-8 promoted cytochrome c and second mitochondria-derived activator of caspases (Smac/Diablo) release from mitochondria and then indirectly activated caspase-9. The mitochondrial and caspase apoptotic pathways are intimately connected. For example, caspase-8 cleaves the cytosolic proapoptotic protein Bid. Bid is a member of the BH3 domain-only subgroup of Bcl-2 family members. This set of proapoptotic proteins shares its only sequence homology within the BH3 amphipathic-helical domain that is essential for killing activity and heterodimerization with other Bcl-2 family members. Upon cleavage, Bid translocates to mitochondrial membranes and binds to Bad, which is another Bcl-2 family protein, and induces release of cytochrome c from mitochondria. Cytochrome c in turn causes Apaf-1 to activate caspase-9 [40] (Figure 6).

Therefore, these suggested that FG020326 might play a dual role in regulating cell death induced by docetaxel (1) by increasing intracellular accumulation of docetaxel and (2) by sensitizing the activation of caspase-8, -9 and caspase-3. Hence there might be other mechanisms for FG020326 reversing MDR except inhibiting P-gp, and next we will explore these mechanisms. Besides, how does P-gp protect from cells apoptosis induced by caspase-8 activated as apical of caspase pathway? This still remains to be seen. It is tempting to speculate that P-gp may protect cells from apoptosis via pumping active caspase-8, -9 or/and caspase-3 out of the cells, by inhibiting caspase activation, by decreasing intracellular ATP or by altering intracellular pH. These all require further study.

## 3. Experimental

### 3.1. Materials

FG020326 was synthesized based on structure-activity relationship as previously described [41] and the purity was more than 98%. Its structure was shown in Figure 1A. RPMI 1640 was purchased from Gibco BRL. Docetaxel, Hoechst 33258 and 3-(4,5-dimethylthiazol-yl)-2,5-diphenyltetrazolium bromide (MTT) were purchased from Sigma Chemical Co. For cell treatments, docetaxel was dissolved in dimethyl sulphoxide (DMSO) and further diluted in culture medium with the final DMSO concentration <0.1%. Mouse monoclonal antibodies (mAbs) against caspase-8 (Ab-3), caspase-3 (E-8) and caspase-9 (F-7) were purchased from Santa-Cruz Biotechnology (Santa Cruz, CA). PARP (c-20) was purchased from Pharmingen (San Diego, CA, USA). HRP-conjugated anti-mouse and anti-rabbit IgG were purchased from Calbiochem (La Jolla, CA, USA).

### 3.2. Cell Lines and Cell Culture

KB and KBv200 cells are human epidermoid carcinoma cell lines. KBv200 cells have a high expression of P-gp, which is one of the most major causes for inducing MDR. P-gp^+ve^ KBv200 cells and their parental sensitive P-gp^−ve^ KB cells were obtained from The Chinese Academy of Medical Sciences, Beijing. KBv200 cells and KB cells were cultured with RPMI 1640 culture medium with 10% fetal bovine serum, benzylpenicillin (100 units/mL), and streptomycin (100 units/mL) at 37 °C in a humidified atmosphere of 5% CO_2_ + 95% air [42].

### 3.3. MTT Cytotoxicity Assay

The cells were collected with trypsin and resuspended to a final concentration of 1 × 10^5^ cells/mL, 0.18 mL aliquots were seeded in 96-well multiplates. The 10 μL of both modulator and anticancer drug (docetaxel) were added after 24 h incubation. After 68 h, 10 μL of 10 mg/mL MTT solution were added to each well, and the plate was incubated for 4 h, allowing viable cells to reduce the yellow MTT into dark-blue formazan crystals, which were dissolved in 150 μL of DMSO. The cell growth inhibition was evaluated by the MTT method on triplicate assays. IC_50_ values were calculated from cytotoxicity curves by Bliss’s method. The degree of resistance was calculated by dividing the IC_50_ for MDR cells by that for parental sensitive cells. The fold-reversal of MDR was calculated by dividing the IC_50_ for cells to anticancer drug in the absence of modulator by that in the presence of modulator [43].

### 3.4. Cell Apoptosis Examined by DNA Fragmentation Analysis

DNA fragmentation was analyzed as described by Herr *et al.* with some modifications [44]. Briefly, cells were harvested and rinsed twice in ice-cold PBS. Final pellets were lysed in 0.3 mL 10 mM Tris-HCl (pH 7.4) buffer containing 25 mM EDTA, 0.5% SDS and 0.1 mg/mL proteinase K (Sigma) and incubated at 37 °C for 12 h. DNA was extracted with an equal volume of phenol/chloroform/isoamyl alcohol (25:24:1) and precipitated with two volumes of ice-cold absolute ethanol and 1/10 volume 3 M sodium acetate. DNA was collected, washed once with 70% ethanol and dissolved in TE buffer (10 mM Tris and 1 mM EDTA, pH 8.0). Samples were incubated with RNase for 1 h at 37 °C. Equal amounts (10~20 μg/well) of DNA were electrophoresed in 1.8% agarose gels impregnated with ethidium bromide (0.1 g/mL) for 2 h at 80 V. DNA fragments in a ladder pattern were visualized by UV transillumination.

### 3.5. Analysis of Cell Apoptosis by Flow Cytometry

Cell apoptosis analysis by flow cytometry was performed as previously described [23]. Briefly, the cells were harvested (trypsinization and centrifugation), fixed with 90% ethanol, and incubated with a staining solution containing 0.2% NP-40, RNase A (30 μg/mL), and propidium iodide (50 μg/mL) in phosphate-citrate buffer (pH 7.2). Cellular DNA content was analyzed by flow cytometry using a Becton Dickinson laser-based flow cytometer. The phase sub-G1 represented the apoptosis. At least 20,000 cells were used for each analysis, and results were displayed as histograms.

### 3.6. Apoptotic Cells Detected by HOECHST 33258 Dye

Cells were cultured with docetaxel in the presence or absence of FG020326 for 48 h, both floating and trypsinized adherent cells were collected, washed with phosphate-buffered saline, fixed with 10% paraformaldehyde for 30 min, and incubated in Hoechst 33258 (Sigma) at room temperature for 30 min (final concentration, 30 µg/mL). Nuclear morphology was examined using fluorescence microscopy with standard excitation filters. Apoptotic cells stained brightly and displayed condensed and fragmented nuclei, normal cells showed an even distribution of the stain throughout the nucleus with flocculated chromatin. To calculate the percentage of apoptotic cells, all cells from four random microscopic fields at 400× magnification were counted [45].

### 3.7. Immunoblot Analysis

The cells were treated with docetaxel in the presence or absence of FG020326 for 48 h. After treatment, whole-cell lysates were extracted with lysis buffer containing 1% Triton-100, 50 mM sodium chloride, 50 mM sodium fluoride, 20 mM Tris (pH 7.4), 1 mM EDTA, 1 mM EGTA, 1 mM sodium vanadate, 0.2 mM phenylmethylsulfonyl fluoride and 0.5% Nonidet P-40. Western blotting was carried out as described previously [46]. In brief, equal amounts of cell lysate (25 μg) were solubilized in 8%~15% SDS-PAGE and transferred onto nitrocellulose membranes. After blocking with non-fat milk, membranes were incubated with the appropriately diluted primary antibody. Then, membranes were incubated with a horseradish peroxidase-conjugated secondary antibody. Proteins were detected by the enhanced chemiluminescence detection system (Amersham, Aylesbury, UK).

### 3.8. Caspase-8 Activity Assay

To further confirm whether the apoptosis induced by docetaxel was involved in caspase-8 dependent pathway, caspase-8 activity analysis was conducted. The cells were cultured with docetaxel of 100 nmol/L in the presence or absence of FG020326 of 5 μM for 12, 24, 48, 72 h, respectively. The assay was performed using the HTS Caspase-8 Activity Assay Protocol (Oncogene). After 1 h incubation at 37 °C, samples were measured using the Fluoroskan Ascent fluorometer (Labsystems) at an excitation wavelength of 390 nm and an emission of 510 nm in a 96-well plate.

### 3.10. Statistical Analysis

Data are means of at least three different experiments ±standard deviation (SD). Student’s *t*-test was used to compare the result. *p* < 0.05 was considered statistically significant.

## 4. Conclusions

In conclusion, FG020326 could reverse in part the apoptotic resistance to docetaxel by sensitizing the activation of caspase-8, -9 and caspase-3. In addition there might be other mechanisms for FG020326 reversing MDR that are tempting to explore.
